# Quality Assessment of YouTube Videos as an Information Source for Testicular Torsion

**DOI:** 10.3389/fpubh.2022.905609

**Published:** 2022-05-18

**Authors:** Gaochen Bai, Xi Pan, Tianxin Zhao, Xiong Chen, Guochang Liu, Wen Fu

**Affiliations:** ^1^Department of Pediatric Urology, Guangzhou Women and Children's Medical Center, Guangzhou Medical University, Guangzhou, China; ^2^Department of Pediatric Surgery, Guangzhou Institute of Pediatrics, Guangzhou Women and Children's Medical Center, Guangzhou Medical University, Guangzhou, China; ^3^Department of Urology, Civil Aviation General Hospital, Civil Aviation Medical College of Peking University, Beijing, China

**Keywords:** testicular torsion, internet, quality, DISCERN, YouTube

## Abstract

**Background:**

Testicular torsion is an acute scrotal disease requiring urgent management, and the COVID-19 pandemic has been demonstrated to lead to poor outcomes for this disease. Presently, many people tend to seek health information *via* YouTube. This study aims to quantitatively assess the quality of English YouTube video content as an information source of testicular torsion.

**Methods:**

In this cross-sectional study, a search was performed with the search term “testicular torsion” on YouTube, and the first 100 videos listed by relevance were selected for our analysis. Duplicate, non-English, videos without audio and surgical videos were excluded. Video features (duration, number of days online, views, likes, comments), source of the video, and author's country were collected. Each video included in the study was assessed using DISCERN and Journal of the American Medical Association (JAMA) Benchmark Criteria. A correlation analysis was performed considering video features, video source, DISCERN scores and JAMA scores.

**Results:**

A total of 66 videos were included and analyzed. The most common video content was general information, including etiology, symptoms, and treatment. The majority of videos were from education and training websites (30%), physicians (23%), and independent users (21%). The mean DISCERN and JAMA scores were 36.56 and 2.68, respectively. According to DISCERN, the quality of video uploaded by physicians was relatively high (*P* < 0.001), and the quality of video uploaded by independent users was relatively low (*P* < 0.001). The JAMA score had no relevance to the video source (*P* = 0.813). The correlation between the video features, DISCERN and JAMA scores was controversial by different assessment methods.

**Conclusions:**

Despite most of the videos on YouTube being uploaded by medical or education-related authors, the overall quality was poor. The misleading, inaccurate and incomplete information may pose a health risk to the viewers, especially during the COVID-19 pandemic. Much effort needs to be undertaken to improve the quality of health-related videos regarding testicular torsion.

## Introduction

Testicular torsion involves twisting the spermatic cord and its contents along with the longitudinal access with resultant ischemia [1]. It accounts for ~10–15% of acute scrotal disease in children, with an incidence rate of 1/4,000 in males younger than 25 years [2]. The torsion of the spermatic cord can reach 180 to more than 720 degrees, resulting in different levels of ischemia and even necrosis in testicular tissue. The viability of testis decreases 6 h after the onset of symptoms [3]. It has been reported that the orchiectomy rate was 42% in boys undergoing surgery for testicular torsion, and ~56.6% of boys receiving salvage orchiopexy had testicular atrophy [4, 5]. As the prognosis of testicular torsion is closely related to the degree and duration of torsion, and the symptoms need to be differentiated from orchitis and epididymitis, it is vital that patients be promptly identified and receive correct management. European Association of Urology (EAU) guidelines recommend early manual detorsion or direct surgical exploration in all suspected patients with testicular torsion [6].

Due to the urgency and harmful outcomes, testicular torsion is challenging for patients or guardians to access early and proper treatment. Testicular atrophy or orchiectomy caused by testicular torsion is not unusual and may affect testicular function and fertility [7, 8]. Some studies found that wait time for manual detorsion or surgery positively correlated with orchiectomy, highlighting the importance and urgency of early identification and intervention for testicular salvage [9, 10]. The absence of knowledge about testicular pathology and the “watch and wait” strategy from adolescents and their parents were also adverse factors preventing timely medical help [11]. Patients or their guardians should have a comprehensive and accurate understanding of the disease. Once symptoms appear, preliminary estimates can be made to avoid the neglect of testicular torsion. On the other hand, they need good quality information to make informed decisions with their physicians.

Since the COVID-19 infection outbreak and global spread, social distancing and reduced travel have been strictly advised to control the spreading of the disease [12]. Usage of medical resources has also been affected, mainly for increased critically ill patients and overcrowding of medical facilities. Reduced number of hospital visits and delays in hospitalization and operations for pediatric patients have been reported due to the parents' hesitancy for symptoms not related to COVID-19, which may lead to more complications and poor outcomes, especially for patients with testicular torsion [13–15]. Such a fearful environment prompts people to access health information and try identifying early symptoms of the disease online.

The internet has developed rapidly and become an essential approach for obtaining and disseminating health information [16, 17]. YouTube is the most popular media search and sharing platform globally, which has been widely utilized to search for and learn health information, especially in young adults [18]. Several studies have reported that people use YouTube as a source of health information to update knowledge, seek help before visiting hospitals, or even purchase healthcare services [19, 20]. However, YouTube may contain misleading or poor-quality information due to the absence of any regulations or restrictions on video content for any uploader [21, 22]. To date, there are many videos about testicular torsion on YouTube, but the literature lacks a quality evaluation of YouTube's content on testicular torsion.

This study aims to quantitatively assess the quality of English YouTube video content as an information source of testicular torsion online.

## Materials and Methods

### Recruitment

In this cross-sectional study, a search was performed on YouTube on March 15th, 2022, with the search term “testicular torsion.” The search history was deleted before searching to reduce any impact on the search results and outcomes. The first 100 results listed by relevance were selected (default YouTube search setting). Duplicate videos, non-English, videos without audio and surgical videos were excluded.

### Collection of Video Features and Source

Video features assessed include total video duration, number of days online, number of views, number of views/day, number of “likes,” number of likes/day, number of comments, number of comments/day, video content type, and author's country. According to the authors, video sources were defined as physicians, patients, education and training websites, news media, medical institutes, and independent users. Video content was classified as general information (etiology, symptoms, and treatment), scrotal ultrasound training, differential diagnosis compared with other scrotal diseases, case discussion, and surgical teaching.

### Assessment of Quality

DISCERN and JAMA Benchmark Criteria were used for quality analyses of the videos [23]. Specialized medical issues related to the disease were based on the EAU guidelines [6].

DISCERN consisted of 16 questions in total, with each question scored from 1 to 5 points. Questions were divided into three parts: reliability (questions 1–8), quality information about treatment options (questions 9–15), and overall score (question 16). The total DISCERN score was calculated by summing up scores over questions 1–15. All videos were divided into five categories based on their total DISCERN score: very poor (<27), poor (27–38), fair (39–50), good (51–62), and excellent (63–75) [24, 25]. JAMA benchmark criteria were used to evaluate online health information reliability, including four criteria (authorship, attribution, disclosure, and currency). Each satisfied criterion counted 1 point, and the maximum possible score was 4 points [26].

Two independent urologists (GB and XP) evaluated all videos. Any discrepancies between reviewers were resolved by discussion with a third author for consensus (GL).

### Ethics Statement

This study focused on the quality assessment of YouTube videos contributed and viewed by the public, so ethics committee approval was not required.

### Statistical Analysis

Statistical analyses were conducted using SPSS software version 26.0 (SPSS Inc., Chicago, IL, USA). Categorical variables were presented as frequency and ratios (%), and continuous variables were presented by mean ± standard deviation (SD) and median (min–max). The Kruskal-Wallis test determined statistically significant differences between more than two groups of any independent variable. Spearman's correlation coefficient was used to evaluate the correlations among variables. A *P* < 0.05 was considered statistically significant.

## Results

### Results of Video Features and Quality Assessment

**Figure 1:**
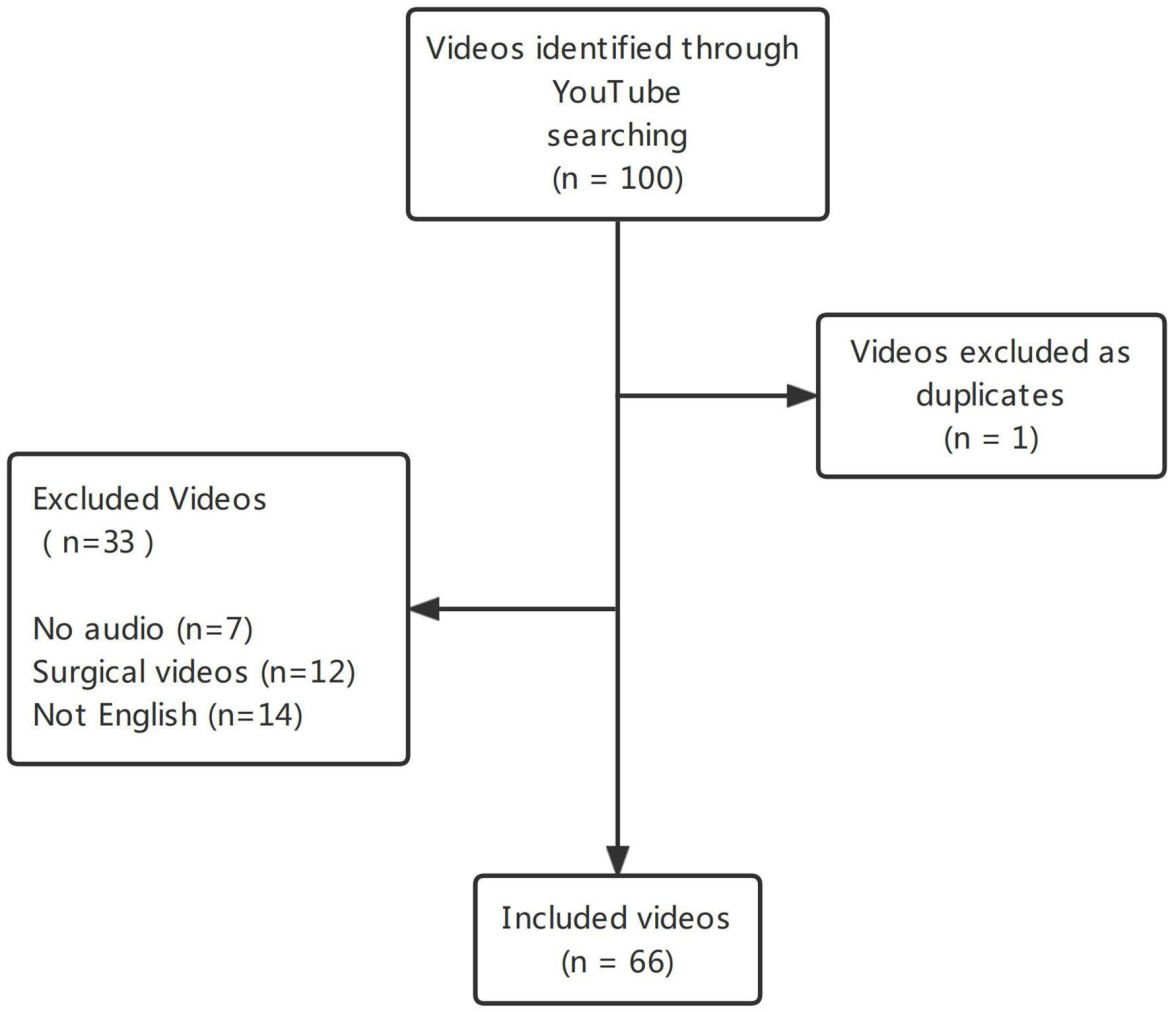
Flowchart of the selection of YouTube videos for analysis.

Among the 100 videos in the initial search were one duplicate video, 7 non-English videos, 12 surgical videos, and 7 videos without audio, resulting in 66 included and analyzed for this study (Figure 1). The 66 video contents, types and features are presented in Table 1. The average score per DISCERN question and percentage satisfying each JAMA benchmark criterion of all videos are summarized in Tables 2, 3. The most common video content was general information about testicular torsion, including etiology, symptoms, and treatment, accounting for 57.6% of the videos. The mean video duration was 457.89 ± 502.54s (range 13–3,261), and the mean number of views was 68406.61 ± 161471.15 (range 30–843,092).

**Table 1:** Characteristics and quality assessments of YouTube videos.

**Video content**	**Number**	**Percentage**
General information (etiology, symptoms, and treatment)	38	57.6%
Scrotal ultrasound training	10	15.3%
Differential diagnosis	9	16.4%
compared with other scrotal diseases		
Case discussion	5	7.6%
Surgical teaching	4	6.1%
Video features	Mean ± SD	Min–max
Duration (s)	457.89 ± 502.54	13–3,261
Number of days online	1475.67 ± 1195.82	8–4,420
Number of views	68406.61 ± 161471.15	30–843,092
Number of views/day	1150.62 ± 8788.11	0.02–71,442
Number of likes	443.06 ± 1072.55	0–6,764
Number of likes/day	13.30 ± 104.02	0–845.5
Number of comments	89.33 ± 228.26	0–1,234
Number of comments/day	0.30 ± 1.60	0–12.88
JAMA score	2.68 ± 0.98	1–4
DISCERN reliability	19.61 ± 6.81	8–33
DISCERN treatment	16.95 ± 7.71	7–33
DISCERN quality	2.62 ± 1.26	1–5
DISCERN total	36.56 ± 13.75	16–66

**Table 2:** Average score per DISCERN question among all included YouTube videos.

		**Question**	**Average score**
Section 1			
	1	Are the aims clear?	3.5
	2	Does it achieve its aims?	3.4
	3	Is it relevant?	3.5
	4	Is it clear what sources of information were used to compile the publication (other than the author or producer)?	1.9
	5	Is it clear when the information used or reported in the publication was produced?	1.8
	6	Is it balanced and unbiased?	2.2
	7	Does it provide details of additional sources of support and information?	1.5
	8	Does it refer to areas of uncertainty?	1.8
Section 2			
	9	Does it describe how each treatment works?	2.2
	10	Does it describe the benefits of each treatment?	2.5
	11	Does it describe the risks of each treatment?	2.2
	12	Does it describe what would happen if no treatment is used?	2.7
	13	Does it describe how the treatment choices affect overall quality of life?	2.5
	14	Is it clear that there may be more than 1 possible treatment choice?	2.2
	15	Does it provide support for shared decision making?	2.9
Section 3			
	16	Based on the answers to all of these questions, rate the publication's overall quality as a source of information about treatment choices.	2.6

**Table 3:** JAMA benchmarks, number, and percentage of YouTube videos.

**JAMA**
**benchmarks**	**Explanation**	**Number**	**Percentage**
Authorship	Authors and contributors, their affiliations, and relevant credentials should be provided.	53	80.3%
Attribution	References and sources for all content should be listed clearly, and all relevant copyright information should be noted.	19	28.8%
Disclosure	Website “ownership” should be prominently and fully disclosed, as should any sponsorship, advertising, underwriting, commercial funding arrangements or support, or potential conflicts of interest.	39	59.1%
Currency	Dates when content was posted and updated should be indicated.	66	100%

**Figure 2:**
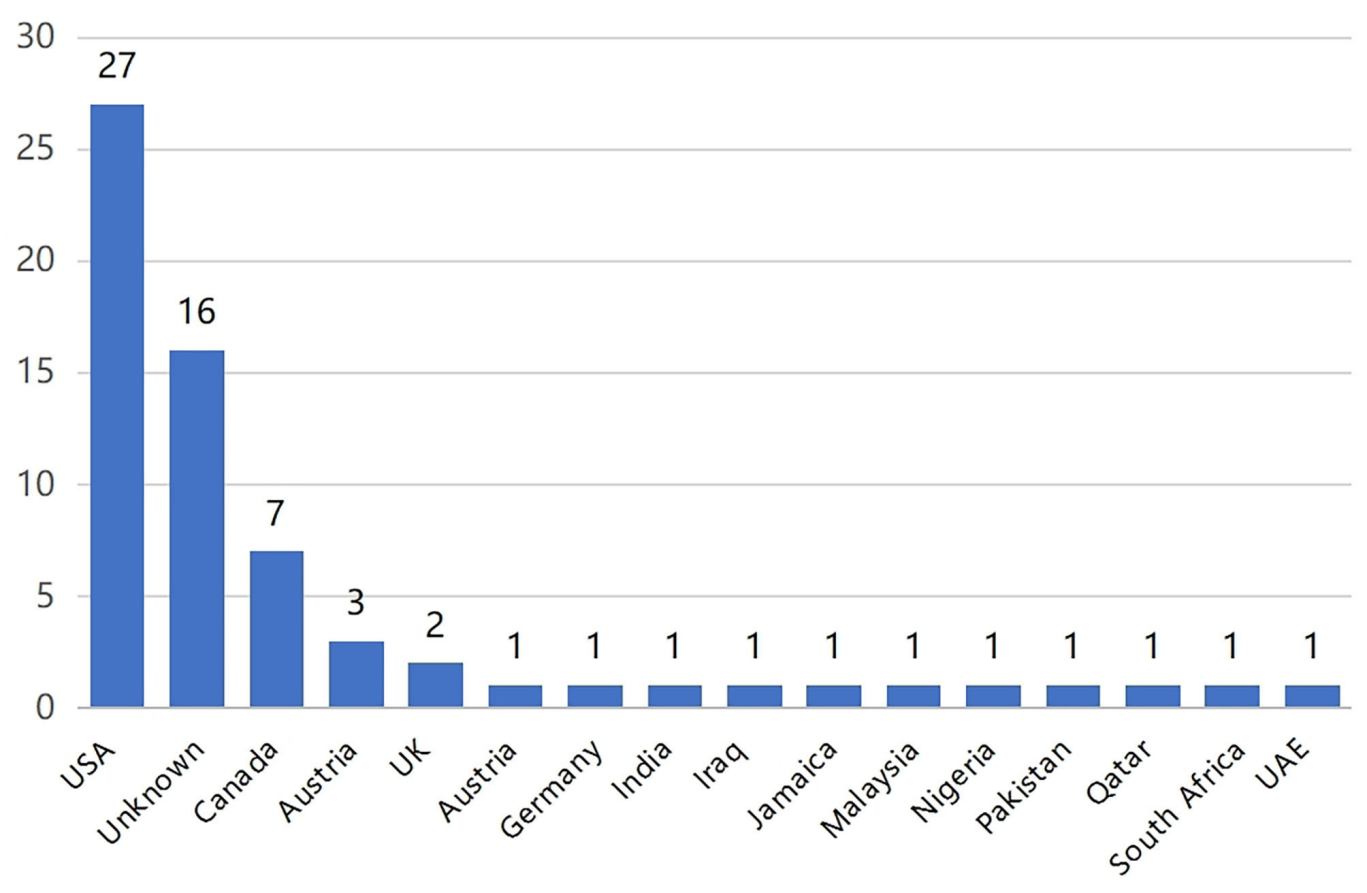
Distribution of authors' countries of YouTube videos.

The mean DISCERN total score was 29.60 ± 9.77 (range 1–4), and the mean DISCERN total score was 36.56 ± 13.75 (range 16–66). Figure 2 shows the distribution of authors' countries, and American authors uploaded the most videos.

### Association of the Source of Videos, Video Features, DISCERN Scores and JAMA Scores

**Figure 3:**
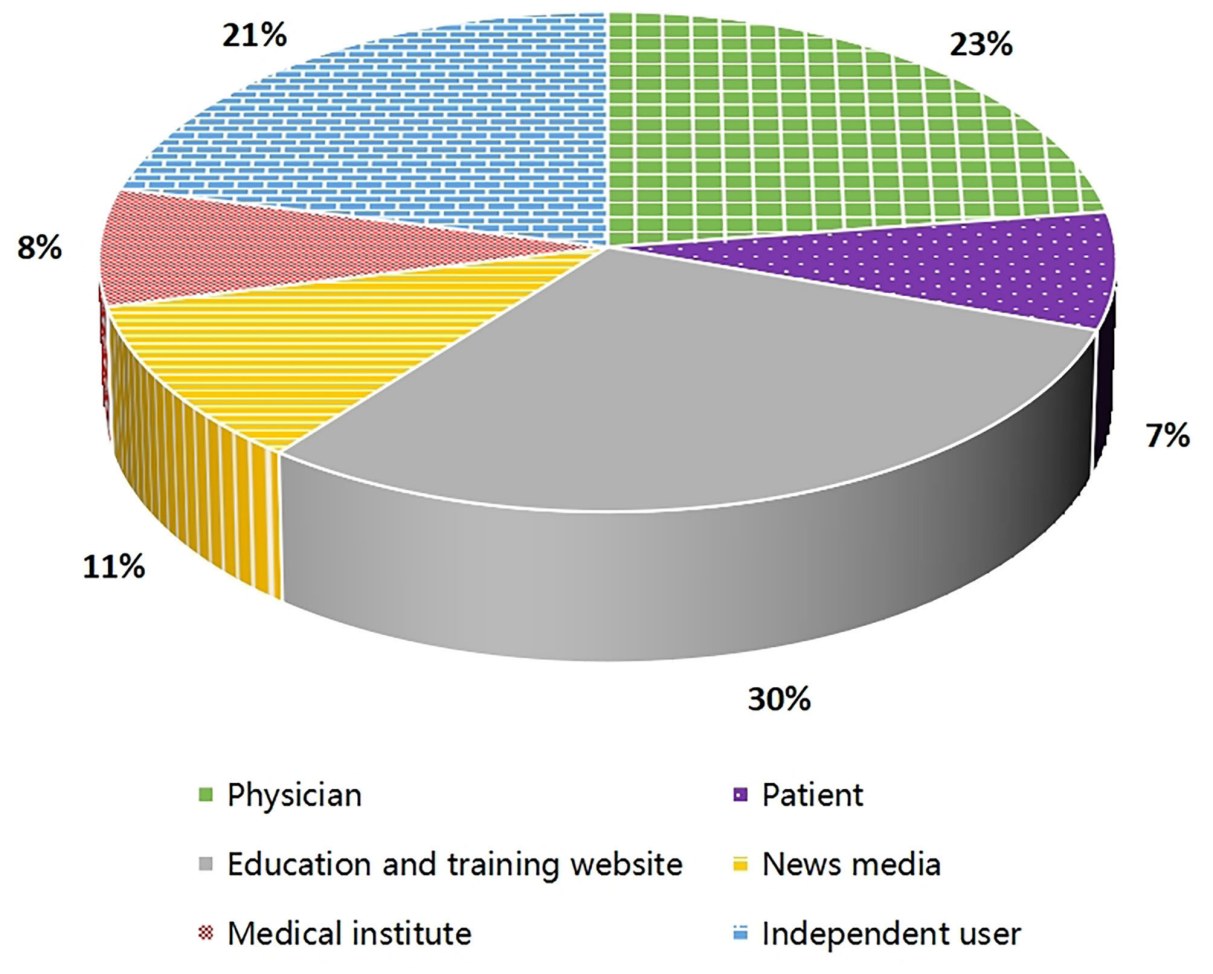
Source of included videos.

The data in Figure 3 shows that the majority of videos were uploaded by education and training websites (30%), physicians (23%), and independent users (21%). The Kruskal-Wallis test showed that the source of videos had a significant association with number of views/day, likes, and likes/day (*P* = 0.006, 0.004 and 0.001, respectively), but no relevance to number of views. Also, reliability scores, treatment scores, quality scores, and total DISCERN scores had a significant association with the source of videos (*P* < 0.001, = 0.002, <0.001 and <0.001, respectively). According to the Bonferroni adjustment, number of views/day, likes, and likes/day were significantly more in videos uploaded by education and training websites than by independent users (*P* = 0.018, 0.008, and 0.006, respectively), and DISCERN reliability scores were significantly higher in videos uploaded by physicians than by patients and independent users (*P* = 0.048 and 0.001, respectively). Additionally, DISCERN total scores were significantly lower in videos uploaded by independent users than by physicians and education and training websites (*P* = 0.001 and 0.006, respectively). The JAMA scores had no relevance to the video source (*P* = 0.813) (see Table 4).

**Table 4:** Video features and quality assessments according to the video source^a^.

**Variable**	**Physician**	**Patient**	**Education and** **training website**	**News media**	**Medical** **institute**	**Independent user**	***P-*value^**b**^**
Number of views	17,980 (122, 230,657)	13,810 (5,057–52,072)	30,331 (150, 843,092)	5,758 (90, 54,367)	6,330 (1,169, 174,227)	1387.5 (30, 69,584)	0.055
Views/day	11.42 (0.27, 74.07)	18.44 (3.49–24.20)	18.10 (0.54, 71,442)^c^	1.38 (0.29, 20.20)	16.82 (1.78, 332.49)	0.75 (0.02, 56.98)	0.006
Number of likes	152 (3, 1,329)	176 (61–727)	203.5 (3, 6,764)^d^	16 (0, 237)	14 (0, 1,119)	5 (0, 590)	0.004
Likes/day	0.13 (0.007, 0.68)	0.21 (0.04–0.44)	0.35 (0.003, 845.5)^e^	0.005 (0, 0.09)	0.08 (0, 2.14)	0.004 (0, 3.91)	0.001
JAMA score	2 (1–4)	3 (1–3)	2.5 (1–4)	3 (1–3)	4 (2–4)	3 (1–4)	0.813
DISCERN reliability	26 (12–33)^f^	14 (8–17)	22 (9–33)^g^	16 (8–22)	21 (19–23)	14.5 (8–21)	<0.001
DISCERN treatment	24 (9–33)	12 (8–17)	19 (7–62)	13 (8–24)	19 (11–29)	10.5 (7–17)^h^	0.002
DISCERN quality	4 (1–5)^i^	2 (1, 2)	3 (1–5)^j^	2 (1–3)	3 (2–4)	1.5 (1, 2)	<0.001
DISCERN total	51 (21–66)	26 (16–33)	43 (17–62)	29 (16–46)	38 (32–49)	25 (16–35)^k^	<0.001

a *Results are presented as median (min–max)*.

b *Kruskal-Wallis test*.

c *Compared with independent user, P = 0.018*.

d *Compared with independent user, P = 0.008*.

e *Compared with news media and independent user, P = 0.021 and 0.006 respectively*.

f *Compared with patient and independent user, P = 0.048 and 0.001 respectively*.

g *Compared with independent user, P = 0.003*.

h *Compared with physician and education training website, P = 0.003 and 0.026 respectively*.

i *Compared with patient and independent user, P = 0.024 and < 0.001 respectively*.

j *Compared with independent user, P < 0.001*.

k *Compared with physician and education training website, P = 0.001 and 0.006 respectively*.

### Evaluation Outcomes of DISCERN Classification

According to DISCERN classifications, 30.3% were “very poor,” 30.3% were poor, 18.2% were “fair,” 18.2% were “good” and 3.0% were “excellent.” There was no statistically significant correlation between DISCERN classification and duration, number of views, likes, comments, views/day, likes/day, comments/day, JAMA scores (see Table 5).

**Table 5:** Distribution of DISCERN classification according to the video source and features.

**Variable**	**Very poor**	**Poor**	**Fair**	**Good**	**Excellent**	***P*-value^**a**^**
Number of	20 (30.3%)	20 (30.3%)	12 (18.2%)	12 (18.2%)	2 (3.0%)	
videos						
Duration (s)	420.65 ± 716.91 (204.5)	364.80 ± 316.83 (283)	585.50 ± 512.68 (331)	467.58 ± 289.04 (381)	937.5 ± 273.65 (937.5)	0.096
Number of views	26,902.70 ± 59,449.27 (4403)	60,246.05 ± 151,868.46 (4814.5)	85,862.33 ± 155,166.84 (9262.5)	140,528.33 ± 272,728.82 (18,900)	27,586.50 ± 33,876.78 (27,586.5)	0.550
Views/day	56.02 ± 213.60 (4.63)	44.52 ± 90.42 (4.14)	63.59 ± 109.10 (6.27)	6,095.08 ± 20,583.04 (29.70)	12.85 ± 13.87 (12.85)	0.251
Number of likes	202.75 ± 392.24 (54.5)	304.25 ± 515.13 (59.5)	427.50 ± 683.01 (18.5)	1132.25 ± 2221.10 (182)	192.50 ± 205.76 (192.5)	0.559
Likes/day	0.39 ± 1.330 (0.05)	0.47 ± 0.95 (0.04)	0.38 ± 0.61 (0.03)	71.33 ± 243.81 (0.23)	0.09 ± 0.08 (0.09)	0.332
JAMA score	3 ± 1 (3)	2.5 ± 0.89 (3)	2.67 ± 1.15 (2.5)	3.17 ± 0.83 (3)	3.5 ± 0.71 (3.5)	0.224
Source of						
the video						
Physician	2	3	2	7	0	
Patient	3	2	0	0	1	
Education and	4	4	6	5	1	
training						
website						
News media	3	2	2	0	0	
Medical institute	0	3	2	0	0	
Independent user	8	6	0	0	0	

a *Kruskal-Wallis test*.

### Correlation Analysis for Any Factors Influencing JAMA and DISCERN Scores

The correlation test showed that DISCERN total scores were significantly positively correlated with video duration (r = 0.335, *P* = 0.006), number of views/day (r = 0.309, *P* = 0.012), likes (r = 0.050, *P* = 0.043), likes/day (r = 0.298, *P* = 0.015) and JAMA score (r = 0.259, *P* = 0.036). The JAMA scores were positively correlated with duration, number of views, views/day, likes and likes/day, but there were no statistically significant correlations (see Table 6).

**Table 6:** Correlation test for the factors influencing JAMA score and DISCERN score.

**Variable**	**JAMA score**		**DISCERN score**	
	**r**	***P-*value^**a**^**	**r**	***P*-value^**a**^**
JAMA score	–	–	0.259	0.036
DISCERN score	0.259	0.036	–	–
Duration (s)	0.108	0.387	0.335	0.006
Number of views	0.034	0.785	0.226	0.068
Views/days	0.106	0.395	0.309	0.012
Number of likes	0.020	0.870	0.050	0.043
Likes/days	0.112	0.369	0.298	0.015

a *Spearman test*.

## Discussion

### Motivation and Meaning of This Study

**Figure 4:**
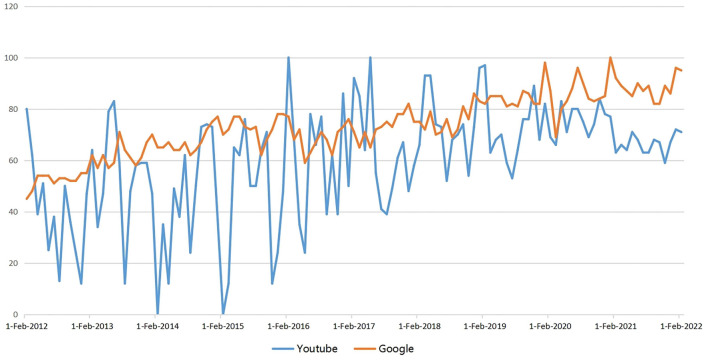
Search trend for the term “testicular torsion” on YouTube and Google website.

Testicular torsion is an acute scrotal disease requiring urgent management. The COVID-19 crisis limits people's access to health information and increases the difficulty for patients to obtain timely treatment [12–15]. As one of the largest and most visited video platforms, YouTube has become an essential channel for health information dissemination, with a user-friendly experience on computers, tablets, and smart mobile phones. The number of searches for “testicular torsion” on the Youtube and Google websites has increased in the past decade (Figure 4). Anyone can upload videos, and health information is constantly updated, so the information provided by Youtube videos may be inaccurate and out of date.

When evaluating the integrity and reliability of videos related to testicular torsion on YouTube, the selection of quality assessment systems directly affects the evaluation results. The DISCERN criteria were developed to enable patients and information providers to judge the quality of information. JAMA benchmark criteria were published to evaluate the quality of internet information on health care. Both have been used to assess the quality of video information on various diseases in previous reports [23, 27, 28]. Some information about these videos was collected to illustrate their fundamental characteristics and correlation with the outcome data. The research contents were discussed, and multiple physicians and researchers formulated strategies.

### Principal Findings

The mean DISCERN and JAMA scores were 36.56/75 and 2.68/4. According to the DISCERN classification, 60.6% of the videos were of very poor or poor quality, and only 3.0% were assessed as excellent quality. References, sources and copyright information were not mentioned in more than two-thirds of videos, and more than one-third of videos lacked prominent and full disclosure. The low score rates reflected the videos' poor integrity and reliability regarding testicular torsion on YouTube. Keelan et al. [29] first found that 38% of analyzed videos objected to immunization but received a higher mean star rating and more views than those supporting immunization. It was worth noting that 45% of these negative videos conveyed messages that contradicted the reference standards. After that, increasing numbers of studies aroused people's concern for the frequently misleading and poor quality of videos on YouTube. Despite these downsides, the increasing popularity of this video-sharing platform prompts more people to use it to disseminate and acquire health information [30].

The majority of videos were uploaded by authors from the United States and Europe. More than half of the shared contents were general information videos containing etiology, symptoms, and treatment. Education and training websites and physicians were the most common source of the videos. In general, these videos had higher DISCERN scores, and tended to be rated as good or excellent quality according to the DISCERN classification, consistent with the results of other similar studies [23, 27, 28]. Moreover, videos from education and training websites seemed to receive more attention than those from independent users. Such results may be because these authors had more professional and systematic knowledge about the disease and focused more on the integrity and reliability of the videos they uploaded. These findings highlight the importance of actively recommending evidence-based health education materials from relatively professional individuals and institutions.

Many users are used to clicking on the videos with higher playback first, hoping for more reliable and comprehensive information from specialized individuals or groups. However, our study found that the most popular videos did not have the highest quality, the highest valued videos were not the most popular videos, and the number of views had no relevance to the video source In addition, the Kruskal-Wallis test showed that the number of views, views/day, likes, likes/day were not correlated with the DISCERN classification, with the correlative analyses illustrating contrary results. Perhaps the artificial classification covered up some critical values. Despite the discrepancy, this interesting result deserves our attention: the public may not always view and trust high-quality health information with little discernment. Furthermore, we found that there were no statistically significant correlations between JAMA scores and recorded or calculated video data. This result reveals that viewers may not care much about the subjects of JAMA Benchmark Criteria, which are indispensable to the integrity and reliability assessment instead.

### Challenges and Solutions

Testicular torsion is one of the most adversely affected diseases during the COVID-19 pandemic [13–15]. More and more people are turning to YouTube for health information, but the overall quality of these videos is poor. Krakowiak et al. [31] found that the mean DISCERN/JAMA score was 28.1 ± 7.9 and 1.1 ± 0.7, respectively, and more importantly, the videos providing misleading information had a higher like ratio. This worrying state of video platforms may easily lead to patient misunderstandings and prevent them from correct choices and timely treatment, especially for acute diseases. Thus, these platforms should be responsible to the public from ethical and legal perspectives. A previous article suggested that a peer-reviewed process during submission may be an ideal solution, but this procedure is cumbersome [32]. One feasible suggestion for eradicating the inaccurate information would be to ask authors to add sources and references in the introduction section of the health-related video, labeling video segments according to the specified standards. At the same time, video platforms can provide specific questionnaires for the views to assess the video quality, and improve the filtering algorithms to prioritize high-quality videos when searching, based on the continuously updated evaluation results. Overwhelmingly, specialists and academic institutions should provide more high-quality, reliable videos that follow clinical practice guidelines to YouTube.

The difference between video materials and materials obtained from internet searches is that video materials are obtained by users passively accepting recommendations from websites, whereas internet materials allow users to seek and identify them actively. High-quality videos from the platforms can support the public's self-education, which will lead to more accurate identification of health information and less adverse impact from poor-quality videos. This is a virtuous circle of promotion.

### Limitations

This study has several limitations. Firstly, the search results on YouTube are dynamic over time, and the data collected and analyzed only represents one point in time. Secondly, despite deleting the search history before searching, the research results may differ according to different geographic locations, user habits, or other unknown algorithm restrictions. Thirdly, the analysis was limited to the first 100 videos for “testicular torsion,” excluding data outside this domain. However, these results are relatively representative because our study aims to reflect the browsing status of ordinary users, with very few considering past the first 100 search results. Fourthly, the function to check the number of dislikes has been removed by YouTube recently, so disliked data and some interaction indexes, including like ratio and video power index (VPI), cannot be included and analyzed in this study. Finally, assessment instruments for video quality are various and constantly updated [33–36], and video quality may vary from different platforms. This study assessed videos from a single platform by two specified instruments, which may lead to biased conclusions.

## Conclusions

YouTube is a popular and indispensable way for the public to learn about testicular torsion. This study is the first report to assess the quality of videos related to testicular torsion on YouTube. The data revealed that despite most of the videos on YouTube being uploaded by medical or education-related authors, the overall quality was poor. The risks of misleading, inaccurate and incomplete information cannot be ignored, especially in the era of the COVID-19 pandemic. Standards for uploading health information videos need to be established to improve the video quality. Video platforms should improve the filtering algorithms to prioritize high-quality videos when searching. Overwhelmingly, specialists and academic institutions should provide more high-quality, reliable videos which follow clinical practice guidelines. Moreover, self-education of the public promoted by high-quality information are also important.

## Data Availability Statement

The raw data supporting the conclusions of this article will be made available by the authors, without undue reservation.

## Author Contributions

GB and XP designed the study, carried out data analysis, and drafted the manuscript. TZ and XC participated in data analysis, collected all relevant data, and assisted in study conception and design. GL and WF conceived the study, participated in its design and coordination, and helped draft the manuscript. All authors read and approved the final manuscript.

## Funding

This research was supported by the Research Foundation of Guangzhou Women and Children's Medical Center for Clinical Doctor and the Guangdong Basic and Applied Basic Research Foundation (No. 2020A1515110796).

## Conflict of Interest

The authors declare that the research was conducted in the absence of any commercial or financial relationships that could be construed as a potential conflict of interest.

## Publisher's Note

All claims expressed in this article are solely those of the authors and do not necessarily represent those of their affiliated organizations, or those of the publisher, the editors and the reviewers. Any product that may be evaluated in this article, or claim that may be made by its manufacturer, is not guaranteed or endorsed by the publisher.

## Abbreviations

JAMA: journal of the american medical association
VPI: video power index.
